# Advances in Laser Linewidth Measurement Techniques: A Comprehensive Review

**DOI:** 10.3390/mi16090990

**Published:** 2025-08-29

**Authors:** Zhongtian Liu, Hao Zheng, Chunwei Li, Zunhan Qi, Cunwei Zhang, Tie Li, Zhenxu Bai

**Affiliations:** 1 Center for Advanced Laser Technology, School of Electronics and Information Engineering, Hebei University of Technology, Tianjin 300401, China; 2 Hebei Key Laboratory of Advanced Laser Technology and Equipment, Tianjin 300401, China; 3 Innovation and Research Institute, Hebei University of Technology in Shijiazhuang, Shijiazhuang 050299, China; 4 Collaborative Innovation Center for Diamond Laser Technology and Applications, Tianjin 300401, China

**Keywords:** linewidth measurement, spectral analysis, Fabry–Pérot interferometry, phase noise

## Abstract

As a key parameter that defines the spectral characteristics of lasers, the precise measurement of laser linewidth is crucial for a wide range of advanced applications. This review systematically summarizes recent advances in laser linewidth measurement techniques, covering methods applicable from GHz-level broad linewidths to sub-Hz ultranarrow regimes. We begin by presenting representative applications of lasers with varying linewidth requirements, followed by the physical definition of linewidth and a discussion of the fundamental principles underlying its measurement. For broader linewidth regimes, we review two established techniques: direct spectral measurement using high-resolution spectrometers and Fabry–Pérot interferometer-based analysis. In the context of narrow-linewidth lasers, particular emphasis is placed on the optical beating method. A detailed comparison is provided between two dominant approaches: power spectral density (PSD) analysis of the beat signal and phase-noise-based linewidth evaluation. For each technique, we discuss the working principles, experimental configurations, achievable resolution, and limitations, along with comparative assessments of their advantages and drawbacks. Additionally, we critically examine recent innovations in ultra-high-precision linewidth metrology. This review aims to serve as a comprehensive technical reference for the development, characterization, and application of lasers across diverse spectral regimes.

## 1. Introduction

Laser, an acronym for laser amplification by stimulated emission of radiation, refers to a coherent light source renowned for its exceptional monochromaticity, directionality, spatial coherence, and high brightness. The theoretical foundation of laser technology traces back to Einstein’s seminal work in 1917 [1], which first postulated the existence of stimulated emission. In 1958, Shawlow and Townes [2] made a groundbreaking contribution by proposing the extension of maser principles to the optical regime, articulating the conceptual framework for achieving monochromatic coherent laser amplification through optical resonator configurations. The advent of practical laser systems materialized in 1960 with Maiman’s pioneering demonstration of the first ruby laser [3,4], which generated a high-purity laser at 694.3 nm, heralding the dawn of laser science. Over subsequent decades, laser technology has undergone exponential evolution, becoming indispensable across diverse industrial and scientific domains. Continuous advancements in critical laser parameters including wavelength [5], output power [6], and spectral linewidth [7,8] have driven transformative progress across multiple disciplines, including precision manufacturing [9,10], life sciences [11,12], defense technologies [13,14], information systems [15], and fundamental research [16,17].

Over the past decades, the generation and precise measurement of laser linewidth have remained central themes in laser science, as linewidth is a fundamental parameter that defines a laser’s temporal coherence and spectral purity. The quest for ultra-narrow linewidths has driven significant progress not only in resonator design and oscillator optimization but also in the development of high-performance optical coatings, ultra-stable reference cavities, and advanced linewidth-narrowing techniques. For example, nonlinear optical processes have played a pivotal role. In particular, third-order nonlinear effects, notably stimulated Raman scattering (SRS) [18,19,20,21,22] and stimulated Brillouin scattering (SBS) [23,24,25], have emerged as pivotal research frontiers. SRS facilitates wavelength generation across the transparency window of laser media [26,27,28], significantly advancing the spectral coverage [29] and power scalability [30] of vortex beams. Notably, in 2025, researchers at Macquarie University demonstrated a novel application of SRS in diamond crystals, whereby temporal fluctuations were converted into coherent lattice vibrations that were rapidly dissipated, resulting in a linewidth compression by over four orders of magnitude [31]. Conversely, SBS enables narrow-linewidth laser emission with ultrashort pulse durations and diffraction-limited beam quality [32,33,34,35,36,37,38,39].

**Figure 1:**
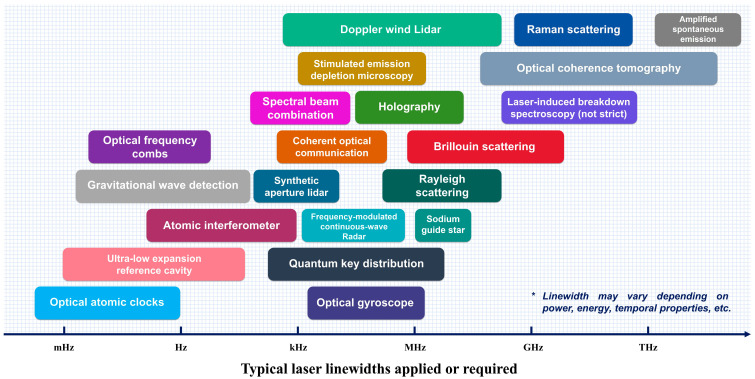
Technological applications of lasers across different linewidth regimes. (Note: linewidth requirements depend on factors such as pulse duration, power level, and application context).

In recent years, narrow-linewidth lasers characterized by superior spectral purity, high peak spectral density, ultralong coherence lengths, and exceptionally low phase noise [40,41] have found indispensable applications across a wide range of fields [42,43,44,45], as schematically illustrated in Figure 1. In optical communication [46,47,48], the deployment of narrow-linewidth lasers has enabled systems to surpass traditional radio-frequency (RF) communication in terms of directionality and carrier frequency, resulting in significantly higher data transmission rates, expanded bandwidth capacity, and enhanced communication security [49,50,51]. Laser communication has further facilitated the implementation of inter-satellite links [52], with prominent satellite constellations such as “Kuiper” [53], “Starlink” [54], and “Xingyun” [55] adopting laser-based transmission as a backbone carrier technology. This paradigm shift signifies the transition of space networks from the radio-frequency era to the laser-dominated era. In coherent optical communication systems [56,57,58,59,60,61,62,63], which utilize the phase coherence of lasers for high-speed and long-distance data transmission, linewidth narrowing is essential for minimizing phase noise at the receiver, thereby reducing the bit error rate (BER) [64] and enhancing overall system performance [65,66,67,68,69,70]. Experimental studies have shown that BER in spaceborne coherent systems is highly sensitive to laser linewidth fluctuations; broader linewidths induce increased phase noise, degrade signal-to-noise ratio (SNR), and result in higher BER. For instance, four-phase shift keying (4-PSK) coherent detection systems necessitate laser linewidths below 100 kHz [71,72,73,74]. In 40 Gbps high-speed optical communication systems, optimal reception performance for advanced modulation formats, including eight-quadrature amplitude modulation (8-QAM), 16-QAM, and 64-QAM, require laser linewidths not exceeding 1.2 kHz [75]. Consequently, lasers with linewidths below 8 kHz are essential to meet stringent BER requirements for high-quality communications [76]. Furthermore, the characterization of laser linewidth demands enhanced precision, as carrier linewidth limitations directly impact the frequency-locking capability of optical phase-locked loops [77,78]. In lidar systems [79,80,81,82,83,84,85,86,87,88,89,90,91,92], particularly coherent lidar, the detection range is inherently limited by the coherence length of the laser source. Employing narrow-linewidth lasers can dramatically extend coherence length, thereby improving measurement range and enabling deployment in large-scale and high-resolution applications. Linewidth narrowing also enhances SNR, which is crucial for precise detection. For instance, lasers with linewidths below 100 Hz [93] are employed in automotive frequency-modulated continuous-wave (FMCW) lidar systems, where wavelength-swept detection enables high-resolution, long-range sensing. In spectroscopy [94,95,96,97,98,99,100], narrow-linewidth lasers with high brightness and tunability allow precise alignment with specific absorption lines, significantly enhancing spectral resolution. This capability supports detailed characterization of atomic and molecular transitions, interaction dynamics, and physical constants, thereby driving progress in high-sensitivity spectroscopic techniques [101,102,103]. For space-based gravitational wave detection [104,105,106,107,108], the stability of laser linewidth governs measurement precision of minute distance variations induced by gravitational waves [109,110]. In the field of atomic clocks [111,112,113,114,115,116,117,118], precise linewidth characterization supports the realization of enhanced temporal and frequency standards, which hold critical importance for satellite navigation and high-speed communication systems. Additionally, narrow-linewidth lasers serve as high-spectral-purity sources in distributed fiber optic sensing [119,120,121,122], where improved linewidth characterization enhances both sensitivity and spatial resolution. These lasers also support more accurate remote sensing [123], enable high-performance quantum sensors [124], and contribute to improved inertial navigation systems [125].

The growing demand for high-performance narrow-linewidth lasers is driven by both fundamental scientific research and the rapid expansion of advanced application frontiers [126,127,128]. Key developments such as external cavity design [129,130,131,132,133,134,135,136,137,138,139], optical phase-locked loop (OPLL) technologies [140,141,142], the Pound–Drever–Hall (PDH) technique [143], self-injection locking (SIL) methods [144,145,146], and distributed feedback (DFB) structures [147,148,149,150,151,152,153,154,155,156,157] have propelled laser linewidths into the kHz regime, with cutting-edge systems achieving Hz-level [158,159] and sub-Hz [160,161] spectral resolutions. Concurrently, precision characterization of ultranarrow linewidth has emerged as a critical challenge [162,163,164,165,166,167,168]. Notably, no single measurement technique is universally applicable across all linewidth regimes. Moreover, the development of linewidth measurement technologies has lagged behind the rapid progress in narrow-linewidth laser sources, creating a critical bottleneck for further advancement. As a result, there is an urgent need for convenient, accurate, and scalable linewidth characterization techniques that can support the continued innovation and optimization of high-performance lasers.

Although a variety of linewidth measurement methods have been proposed in the literature, a systematic and comparative evaluation of their principles, performance limits, and practical constraints remains lacking. This review addresses this gap by summarizing and analyzing existing laser linewidth measurement techniques, organized by applicable linewidth regime from broad to ultranarrow.

## 2. Basic Concept of Linewidth

### 2.1. What Is Linewidth?

Laser radiation is distinguished from conventional light sources by its exceptional coherence properties, encompassing both spatial and temporal coherence. Spatial coherence quantifies the phase correlation across transverse wavefront dimensions during beam propagation, serving as the prerequisite for achieving collimated beam characteristics. Temporal coherence describes the phase relationship of the beam at different time points along the propagation direction, which is proportional to the monochromaticity of the laser. Temporal coherence can be characterized by linewidth.

**Figure 2:**
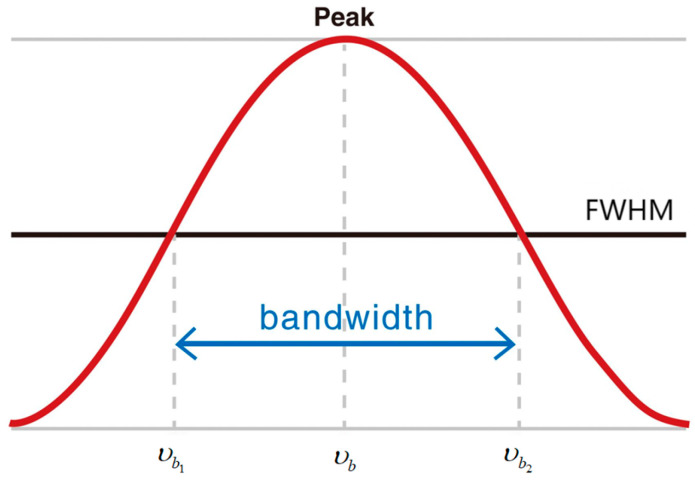
Definition of Laser Linewidth Based on FWHM.

The operational dynamics of laser systems are inherently influenced by dual noise mechanisms: quantum noise stemming from spontaneous emission [2,169,170] and classical noise perturbations induced by environmental factors including mechanical vibrations and thermal instabilities [171,172,173]. This means that in addition to the intrinsic spectral width of the laser with a certain width [174], the actual output spectral line is further broadened, which is the root cause of the existence of the laser linewidth. Quantitatively, linewidth is defined as the full width at half maximum (FWHM) of the optical power spectrum. This refers to the spectral width measured at 50% of the peak intensity, as schematically illustrated in Figure 2.

In 1955, Gordon et al. [174] established a theoretical framework for ammonia molecular beam-driven microwave amplifiers. Through a classical power-balance analysis, they derived the fundamental linewidth expression for microwave amplification systems, as follows:

(1) $$\upsilon_{t}\approx \frac{kT{\left(\Delta \omega \right)}^{2}}{P}$$

where *kT* represents the thermal bath’s spectral power density maintaining equilibrium in the microwave amplifier prior to beam activation, Δ*ω* denotes the molecular emission bandwidth, and *P* corresponds to the emitted power. Both Δ*ω* and *υ_t_* contribute to the FWHM.

Schawlow and Townes proposed the adaptation of Equation (1) to optical regimes [2,175]. The Schawlow–Townes formula can be obtained by simply replacing *kT* with *hυ*, which corresponds to the spectral power density of one photon in each mode, as follows:

(2) $$\upsilon_{t}=\frac{h\upsilon {\left(\Delta \omega \right)}^{2}}{P}$$

This expression forms one of the cornerstones of modern laser physics, highlighting the intrinsic relationship between spontaneous emission and spectral purity.

To account for the dynamics of practical laser systems, Haken [176], Lax [177], and Scully [124] independently proposed a generalized linewidth expression for four-level lasers operating above threshold, now known as the Haken–Lax–Scully formula:

(3) $$\upsilon_{t}=\frac{h\upsilon {\left(\Delta \omega_{\min}\right)}^{2}}{2P}$$

where Δ*ω_min_* refers to the smaller of the natural emission bandwidth and the cavity bandwidth. Experimental measurements by Manes [178] confirmed the general form of this equation, which also accommodates the effects of inhomogeneous broadening.

In essence, laser linewidth is fundamentally determined by the spontaneous emission rate of the gain medium and the structural properties of the resonant cavity [159]. As long as the gain medium is based on the amplification of stimulated emission laser, the lifetime of the upper energy level is limited, and spontaneous emission cannot be avoided. The vibration of the vibration source causes low-frequency noise, and the cavity length disturbance that this causes leads to optical frequency drift, thereby broadening the spectral linewidth. Consequently, linewidth serves as a direct proxy for phase noise evaluation, where narrower linewidth correlate with enhanced frequency stability and suppressed phase fluctuations [179]. Given the multifaceted origins of linewidth variations, ranging from dominant quantum effects to subtle technical noise sources, the development of refined metrological techniques has become imperative for validating linewidth control strategies.

### 2.2. How to Measure Linewidth?

**Figure 3:**
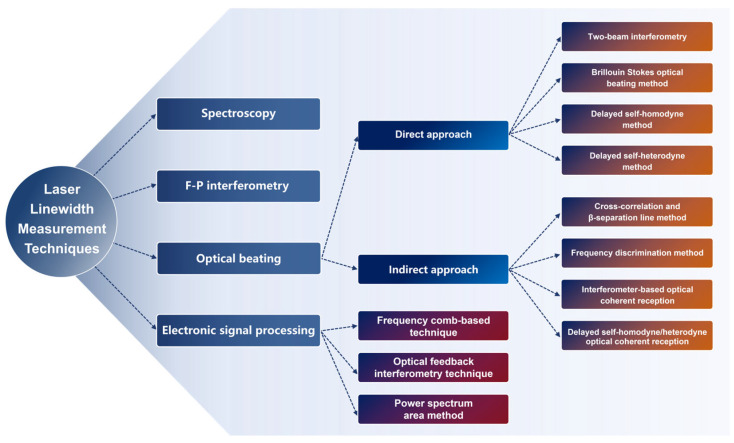
Overview of laser linewidth measurement techniques across different linewidth regimes.

The advancement of laser linewidth measurement techniques has closely paralleled the progress in linewidth compression technologies. As illustrated in Figure 3, a variety of methods have been developed to meet the demands across different linewidth regimes.

For GHz-level linewidths, the spectroscopic method, a relatively mature commercial technique, utilizes high-resolution optical spectrum analyzers (OSAs) to directly determine the FWHM of the laser emission spectrum [180,181,182,183,184,185]. In the MHz regime, Fabry–Pérot (F-P) interferometry provides another effective approach for linewidth characterization [186,187]. However, as laser linewidths continue to narrow, these methods face increasing limitations. The spectroscopic approach is fundamentally constrained by the resolution limits of OSAs, while F-P interferometry becomes increasingly sensitive to cavity length fluctuations and noise floor interference, ultimately rendering both methods inadequate for the precise measurement of ultra-narrow linewidths [188,189,190]. The ongoing refinement of laser frequency stabilization [44,191] and mode-selection techniques [192] has driven significant advancements in narrow-linewidth laser metrology. The laser linewidth can be obtained not only directly by the laser power spectrum, but also indirectly by the measurement of phase noise. Due to the relative ease of acquiring the power spectrum and its intuitive representation of linewidth characteristics, optical beating methods have gained widespread adoption. These techniques are currently capable of resolving linewidths on the order of kilohertz.

In this review, we focus on four representative methods for directly measuring linewidth based on the signal power spectrum: two-beam interferometry, the Brillouin Stokes optical beating method, delayed self-homodyne, and delayed self-heterodyne techniques. While phase-noise-based linewidth measurement offers higher precision, it often involves more complex mathematical modeling and algorithmic processing. Finally, we summarize and provide perspectives on the future development of advanced linewidth measurement techniques, highlighting the need for greater accuracy, robustness, and ease of implementation in emerging applications.

## 3. Spectroscopic Method

**Figure 4:**
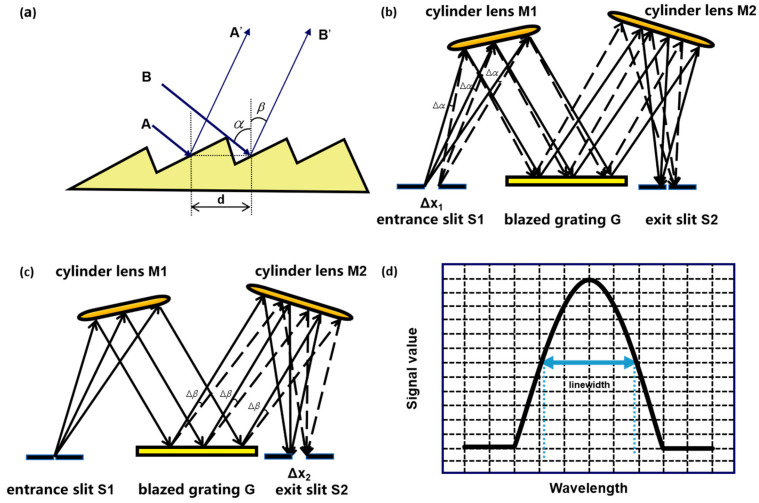
Schematic and components of a grating spectrometer. (**a**) Working principle of a blazed grating. (**b**) Incident slit with defined width. (**c**) Exit slit with defined width. (**d**) Measured laser spectrum with linewidth determined by FWHM.

Laser linewidth can be determined by analyzing the optical spectrum of the emitted light. To achieve this, the spectral information of the laser must first be acquired. Optical gratings exploit their periodic structure to produce constructive interference at specific angles, thereby spatially dispersing different wavelengths. Figure 4a illustrates the operating principle of a blazed grating. Two incident light rays, originating from points A and B, are diffracted by the grating and produce rays AA′ and BB′. In this schematic, *d* denotes the distance between adjacent grooves (i.e., the grating period), which is also the spacing between the corresponding diffracted rays. The angles α and β represent the incident and diffraction angles, respectively. The relationship governing spectral dispersion is described by the grating equation, as follows:

(4) d(sinα+sinβ)=mλ

where *m* indicates the diffraction order (*m* = 0, ±1, ±2, ……), and *λ* is the wavelength of the incident laser.

As shown in Figure 4b, a typical grating spectrometer fundamentally consists of an incident slit (S1), collimating mirror (M1), blazed grating (G), focusing mirror (M2), and exit slit (S2). Polychromatic light entering through S1 is collimated by M1 and projected onto the grating, where wavelength dispersion occurs to generate parallel beams at distinct diffraction angles. M2 focuses a specific wavelength onto S2, and the photodetector (PD) behind S2 records the output signal intensity corresponding to different grating rotation angles. By rotating the grating using a precision rotation stage, full-spectrum scanning is achieved, yielding the spectral intensity distribution as illustrated in Figure 4d. The FWHM of the resulting spectral peak represents the linewidth of the measured laser.

The resolution of a grating spectrometer is primarily determined by three instrumental parameters: the effective focal length, the dispersion characteristics of the grating, and the widths of the entrance and exit slits. While the effective focal length and grating dispersion are intrinsic to a given spectrometer design and cannot be modified, the slit widths are adjustable parameters that must be optimized based on experimental requirements.

### 3.1. Effect of Incident Slit Width on Spectral Linewidth

For the theoretical analysis of the effect of incident slit width on the measured spectral line width, it is assumed that the incident beam is an ideal monochromatic wave, and the width of the exit slit is neglected. As shown in Figure 4b, when the incident slit width of the spectrometer is Δ*x*_1_, each point on the slit can be regarded as a luminous point, and the angular width of the incident angle of the beam incident on the grating is Δ*α*. Since the slit is located on the focal plane of the cylindrical mirror, the value of Δ*α* can be expressed as follows:

(5) $$\Delta \alpha =\frac{\Delta x_{1}}{f_{FL}}$$

By differentiating both sides of Equation (4) with respect to the grating incident angle and substituting Equation (5) into the result, the measured spectral line width caused by the slit width can be expressed as follows:

(6) $$\Delta \lambda =\frac{d\cdot \Delta x_{1}}{mf_{FL}}\cdot \cos\alpha$$

where *f_FL_* denotes the focal length of the cylinder lens.

From Equation (6), it can be observed that the measured spectral line width at this point is proportional to the incident slit width and inversely proportional to *f_FL_*.

### 3.2. Effect of Exit Slit Width on Spectral Line Width

As shown in Figure 4c, when the incident slit width of the spectrometer is $\Delta x_{2}$, the corresponding beam emitted from the grating and passing through the exit slit has an angular width of Δ*β*. By analogy, the measured spectral line width Δ*λ* caused by the exit slit width $\Delta x_{2}$ can be expressed as follows:

(7) $$\Delta \lambda =\frac{d\cdot \Delta x_{2}}{mf_{FL}}\cdot \cos\beta$$

It can thus be seen that the spectral line width is proportional to the exit slit width and inversely proportional to *f_FL_*.

### 3.3. Effect of Slit Width on Spectrometer Resolution

The resolution of a spectrometer is a parameter that indicates its ability to separate two spectral lines with extremely close wavelengths. It is defined as follows:

(8) $$R=\frac{\lambda }{\Delta \lambda_{s}}$$

where *λ* is the wavelength of the light wave, and Δ*λ_s_* is the resolving limit of the spectrometer. As discussed in Section 3.1 and Section 3.2, Δ*λ_s_* is proportional to the slit width; thus, the resolution *R* is inversely proportional to both the exit slit width and the incident slit width [193].

In general, reducing the widths of the incident and exit slits is beneficial for improving the resolution of the spectrometer. However, narrowing the incident and exit slits weakens the signal intensity, and excessively small slit widths may render the signal undetectable by photodetectors. Therefore, when using a spectrometer to measure laser linewidth, the slit widths should be minimized as much as possible under the premise that the optical signal can be detected by the photodetector, to improve the measurement accuracy of the laser linewidth.

Early studies adopted multi-spectrometer collaborative measurement to overcome single-device limitations. H. Liu et al. [194] theoretically analyzed the principle of combined two-spectrometer laser linewidth measurement, derived the dispersion rate formula, and enhanced optical path dispersion via their joint use to increase spectral separation. Experimental results showed this method improved He-Ne laser FWHM measurement accuracy by at least five orders of magnitude over single spectrometers, verifying multi-device error complementation potential and offering a cost-effective scheme for resource-limited labs, though performance remains constrained by hardware’s intrinsic physical properties.

Recent advancements have witnessed the convergence of optical encoding and deep learning algorithms. Liu et al. [195] pioneered a dual-phase framework where optical devices encode incident spectral information and reconstruction algorithms decode it, proposing an adaptive deep learning algorithm to mitigate distortions induced by filter array imperfections.

This method, grounded in the principle of grating dispersion, enables laser linewidth determination through spectral feature analysis while simultaneously acquiring wavelength and intensity parameters, thus supporting real-time observation of spectral dynamics. The spectrometer’s free-space optical interface facilitates direct beam collimation and injection, significantly improving operational efficiency [196]. State-of-the-art diffraction grating spectrometers now achieve remarkable performance metrics, including 5 pm wavelength resolution, 5 pm wavelength accuracy, 65 dB dynamic range, and 80 dB stray light suppression ratio. These advancements enable unprecedented precision in spectral characterization, particularly in applications demanding ultra-narrow linewidth analysis, thereby unlocking new potential for high-resolution spectroscopic investigations [197].

Nevertheless, despite this operational simplicity, different spectrometers have been designed to cover a specific wavelength range. Therefore, lasers with wavelengths beyond this range cannot be measured as they cannot be effectively separated or detected. Furthermore, due to limitations such as the resolution being constrained by the slit width, the difficulty in precisely controlling incident and diffraction angles, and potential overlap of spectral lines from different orders, spectrometers can only measure laser linewidths at the GHz order of magnitude or above.

## 4. F-P Interferometry

F-P interferometry is a laser linewidth measurement technique based on multi-beam interference principles [198,199]. Two configurations are commonly employed: the F-P etalon (with constant cavity length) and the F-P scanner (with tunable cavity length) [200].

The F-P etalon functions as an optical resonator comprising two high-reflectivity parallel surfaces (glass or quartz plates) separated by a medium to form a resonant cavity of length [201,202]. Typical implementations include air-gap etalons and solid fused-silica etalons [203]. The F-P scanner enhances interferometric sensitivity by integrating auxiliary optics such as focusing lenses and polarizers, with cavity designs adopting confocal or quasi-confocal configurations for improved beam alignment [204]. While both variants effectively measure continuous-wave laser linewidth, the F-P etalon suffers from limited sampling rates for low-repetition-rate pulsed lasers, whereas the F-P scanner configuration eliminates pulse frequency constraints, making it ideal for pulsed laser characterization [205].

**Figure 5:**
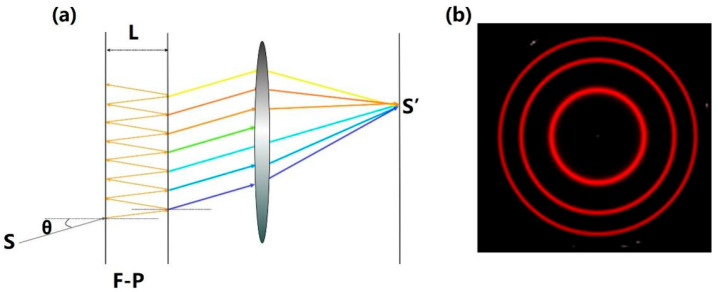
(**a**) Optical path diagram of the F-P interferometer. (**b**) Recorded false-color image of an interference pattern generated using the F-P etalon [206]. L: cavity length of F-P interferometry.

As illustrated in Figure 5a, monochromatic light incident at angle *θ* undergoes multiple reflections and transmissions at the coated surfaces, generating parallel reflected/transmitted beams. When the cavity satisfies the resonance condition (L is the integer multiple of half wavelength), standing waves induce constructive or destructive interference. Collimating the transmitted beams via a convex lens produces a concentric ring pattern on the focal plane S’, as shown in Figure 5b. The interferometric ring pattern is then converted into spectral data through coordinate transformation within the interference domain, followed by Gaussian fitting to extract the interferometric linewidth.

For the F-P interferometer, its spectral resolution is typically characterized by the parameter finesse. Higher finesse implies that the interferometer can generate extremely sharp resonance peaks, enabling it to more readily distinguish closely spaced transmission peaks; thus, high finesse corresponds to high spectral resolution. In practice, the total finesse *F_t_* of an F-P interferometer system is influenced by multiple factors and can be expressed as follows [207]:

(9) $$\frac{1}{F_{t}}=\frac{1}{F}+\frac{1}{F_{Q}}+\frac{1}{F_{i}}$$

where *F* denotes the reflectivity-limited finesse of the mirror, *F_Q_* represents the quality-limited finesse originating from the mirror surface, and *F_i_* accounts for the illumination-limited finesse induced by the mirror’s illumination conditions (including beam alignment and beam diameter).

Most F-P interferometers are designed with carefully engineered reflective coatings such that, over the entire operating wavelength range and under proper illumination conditions, the reflectivity-limited finesse *F* dominates the total system finesse *F_t_* (where a higher reflectivity increases *F*, thereby enhancing the spectral resolution). Regarding the other two finesse components, *F_Q_* characterizes the symmetric broadening of spectral lines induced by microscopic irregularities on the mirror surface. These irregularities introduce random optical path differences across different regions of the mirror, leading to blurred broadening of the interference spectrum due to phase incoherence. In contrast, *F_i_* degrades the resolution as the beam diameter increases or the input beam undergoes misalignment. When the finesse is limited by *F_i_*, the measured spectral line shape exhibits asymmetry.

The performance breakthrough of high-finesse fiber Fabry–Pérot scanning interferometers (FFPSI) originates from the synergistic optimization of finesse and spectral resolution. Since the FFPSI employs a built-in single-mode fiber cavity, it is free from diffraction loss or imperfect spatial mode matching that are inherent in bulk devices. Therefore, the FFPSI can be extended to longer cavity lengths, enabling direct high-resolution measurement of narrow laser linewidths. Early advancements involved constructing high-precision FFPSI systems with 10–40 cm cavity lengths, achieving a resolution of 500 kHz and finesse exceeding 500, representing an order-of-magnitude improvement over conventional designs [208]. While this progress solidified FFPSI’s dominance in static spectral analysis, its dynamic measurement capability remained constrained by scanning rate and cavity length limitations. Subsequent efforts targeting dynamic applications focused on optimizing long-cavity FFPSI scanning rates. Kevin Hsu et al. [209] improved the resolution of FFPSI by increasing the cavity length and reducing the scanning rate. They tuned the FFPSI by adjusting the resonant cavity length via a piezoelectric transducer platform, achieving a resolution of approximately 8 kHz for the long-cavity FFPSI at a scanning rate of about 3 ms per free spectral range. Although the measurement device was sensitive to environmental interferences such as ambient temperature drifts, acoustic waves, and mechanical vibrations, thus requiring appropriate shielding, this achievement provides a valuable reference for developing long-cavity FFPSI into an effective, low-cost, and lightweight instrument that can be directly applied to high-resolution spectroscopic research. The extension of FFPSI applications to pulsed laser characterization introduced new challenges in response dynamics. Xue et al. [210] systematically investigated the differential response mechanisms to continuous-wave and pulsed lasers, demonstrating FFPSI’s capability to measure pulse laser linewidths to 100 MHz. However, conventional interferometry exhibited critical limitations in low-repetition-rate short-pulse (LSL) measurements due to convolutional artifacts and stability-dependent errors. Addressing these constraints, Hun Xuanning et al. [206,211] employed a technique combining a Fabry–Pérot etalon with a complementary metal oxide semiconductor (CMOS) beam profiler. They accurately localized the center of the interference pattern via the Hough transform algorithm, followed by the application of a pixel rotation algorithm to convert the two-dimensional interference pattern into one-dimensional spectral information. This significantly increased the number of effective data points, improved spectral resolution, and reduced the error induced by pixel size to below 1 MHz. Subsequently, they mitigated the error induced by transmission spectrum width (TSW) through deconvolution processing, thereby achieving accurate measurement of the linewidth of short pulses at low repetition rates. This methodology transformed hardware limitations into algorithmic optimization opportunities, drastically reducing measurement time and eliminating stringent laser stability requirements.

For the F-P interferometer, increasing the reflection coefficient enhances *F*, enabling the interferometer to generate sharper resonance peaks and thereby improving spectral resolution. However, this enhancement is constrained by the GHz-scale free spectral range (FSR) of the F-P interferometer, which limits its minimum resolvable frequency difference to the MHz regime. Conversely, lower reflectivity reduces *F*, broadening the resonance peaks and degrading resolution. A critical balance must be maintained between spectral resolving power and net light-gathering efficiency. A pragmatic compromise involves enlarging the mirror aperture until spectral resolution diminishes by 70% [207]. These reasons together lead to the fact that the F-P interferometer can only measure the laser linewidth of MHz magnitude.

## 5. Optical Beating Method

Due to the limitation of resolution, the spectrometer can only measure the linewidth of GHz magnitude, while the linewidth limit detected by F-P interferometry is in the order of MHz [212]. For laser linewidth in the kHz regime and below, the optical beating method emerges as the dominant approach, where precise acquisition and analysis of beat frequency signals are critical. Two optical beams with distinct frequencies and wavelenghs (λ_1_ and λ_2_) are coherently combined and incident on a PD. The PD’s nonlinear response generates a beat signal at the frequency difference, expressed as follows:

(10) $$f_{m}=\frac{\lambda_{1}-\lambda_{2}}{{\lambda_{1}}^{2}}$$

where *f*_m_ presents the difference of the laser frequencies.

**Figure 6:**
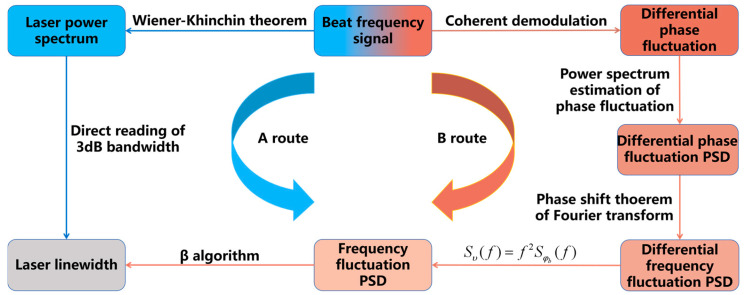
Relationship among beat frequency signals processed by different methods.

The acquired beat signal necessitates sophisticated numerical processing, as systematically outlined in Figure 6, which delineates two distinct analytical workflows and their interrelationships. The data acquisition of beat frequency in time domain is the entry point to obtain the center of linewidth. Route A employs the Wiener–Khinchin theorem [213], wherein autocorrelation of the temporal beat signal is computed prior to Fourier transformation, yielding the laser’s PSD from which the linewidth is directly extracted. In contrast, Route B leverages β-separation theory [214], deriving the linewidth through the intrinsic relationship between phase noise, frequency noise, and linewidth. Since the signal power spectrum contains more intuitive linewidth information and is relatively easy to obtain, most of the linewidth measurement experiments are based on Route A.

### 5.1. Laser Linewidth Measurement Based on the Signal Power Spectrum (Direct Measurement)

**Figure 7:**
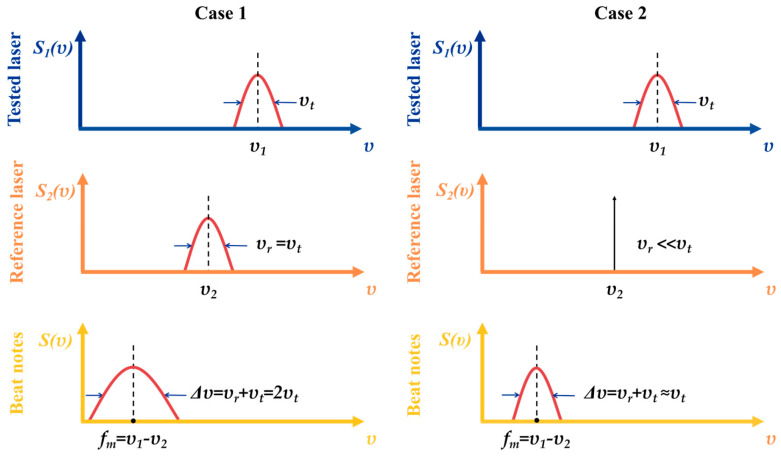
Principle of optical beating method [215] (where *S*_1_(*υ*) is the PSD of the tested laser, and *S*_2_(*υ*) is that of the reference laser).

The method for measuring laser linewidth based on the signal power spectrum is characterized by its simplicity and efficiency, and has been widely applied in practical operations. It corresponds to Route A in Figure 7. When two incoherent lasers with Lorentzian line shapes are used, their beat frequency signal retains a Lorentzian line shape, and the PSD of the beat frequency signal can be expressed as follows:

(11) $$S(\upsilon )=\frac{\Delta_{\upsilon }}{2\pi [{\left(\upsilon -f_{m}\right)}^{2}+{\left(\frac{\Delta_{\upsilon }}{2}\right)}^{2}]}$$

(12) $$\Delta_{\upsilon }=\upsilon_{t}+\upsilon_{r}$$

where Δ*_υ_* is the linewidth of the beat frequency power spectrum, *υ_t_* is the linewidth of the tested laser, *υ_r_* is the linewidth of the reference laser, and *f_m_* is the frequency difference between the two lasers mentioned above.

Linewidth measurement based on the optical beating method typically involves two cases, as shown in Figure 7. When the reference laser and the tested laser have identical linewidth, the linewidth of the beat frequency signal becomes twice that of the tested laser (Case 1). This method is suitable for measuring arbitrary linewidth, but its drawback lies in the complexity of acquiring, measuring, and calibrating the reference laser. When the linewidth of the reference laser is significantly smaller than that of the tested laser and can be neglected, the linewidth of the beat frequency signal approximates that of the tested laser (Case 2). This approach can measure the linewidth of most lasers but struggles with precise measurements of narrow linewidth.

Depending on the specific characteristics of the tested laser, either of these two schemes can be flexibly selected to achieve optimal measurement results. This section highlights four measurement methods based on the two cases, including two-beam interferometry, the Brillouin Stokes optical beating method, the delayed self-homodyne method, and the delayed self-heterodyne method.

Two-beam interferometry, based on Case 1, involves coupling two optical signals with closely matched wavelengths after they pass through an isolator. The intermediate-frequency electrical signal generated from the coupling is then detected by a PD. Finally, an electrical spectrum analyzer (ESA) is used to process the detected signal, enabling the calculation of the laser linewidth.

Unlike two-beam interferometry, the Brillouin Stokes optical beating method (based on Case 2) eliminates the need for an additional reference laser. It uses narrow-linewidth second-order Stokes light generated in a fiber resonator as the reference for beating with the tested laser. A resonant tracking circuit (RTC) and piezoelectric ceramic (PZT) are used to control the fiber length, ensuring the pump light resonates in the fiber resonator.

To avoid the stringent environmental requirements of the two-beam interferometry and overcome the linewidth measurement range limitation of the Brillouin Stokes beating method, the delayed self-homodyne method has emerged, featuring a simple structure, wide measurement range, and low optical transmission loss. Based on Case 1, this method processes the tested laser using an unbalanced Mach–Zehnder interferometer (UMZI) [216].

To steer clear of the rigorous environmental demands of two-beam interferometry and get around low-frequency interference in the delayed self-homodyne method, Okoshi et al. [217] proposed the delayed self-heterodyne method, achieving a resolution of 50 kHz. Subsequently, P. Gallion [218] conducted a detailed derivation of its basic principles. Based on Case 1, this method uses a long delay fiber to create a time delay much longer than the coherence time of the original beam, rendering the two beams incoherent during beating. The spectrum of the beat signal is typically fitted with a Lorentzian function, facilitating linewidth parameter measurement [219]. Furthermore, the system incorporates an acousto-optic modulator (AOM) into the system, shifting the laser signal’s center frequency to a high-frequency region to avoid interference from environmental noise near the zero frequency.

### 5.2. Linewidth Measurement Based on Phase Noise (Indirect Measurement)

In 1986, L.E. Richter et al. [220] theoretically analyzed the laser linewidth measurement principle of this method, proposing that the beat signal exhibits a perfect Lorentzian line shape only when the delay fiber length exceeds six times the coherence length of the laser under test. This results in a 1590 km fiber being required to measure a 100 Hz linewidth [221], and even for 10 kHz linewidths, the delay fiber would still be tens of kilometers long [222]. However, long fibers exacerbate optical power loss and amplify the effects of factors such as Rayleigh scattering [223,224], spectral drift [225], and 1/f noise, causing additional spectral broadening [7,226] and measurement errors [227]. Additionally, high-power lasers in long fibers are prone to SBS, resulting in the conversion of pump energy into Stokes wave and acoustic wave energy, which exacerbates transmission loss and hinders signal detection by the PD. To address these limitations, researchers have proposed various improved schemes for the delayed self-heterodyne method. Table 1 presents the measurement devices, their descriptions and main features, and some references.

**Table 1:** Four direct measurement methods [228,229,230,231,232,233,234,235,236,237,238,239,240,241,242,243,244,245,246,247,248,249,250,251,252,253,254,255,256].

	Measuring Equipment	Description	Case	Main Hallmarks	Reference
Two-beam interferometry	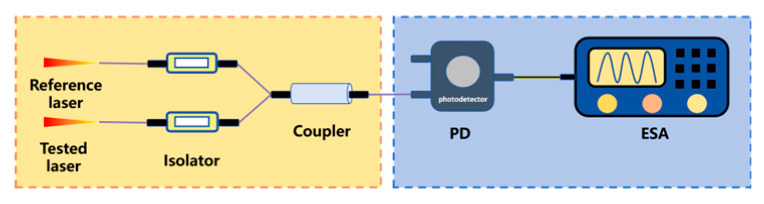	This method determines the laser linewidth by interfering the test laser with a stabilized reference laser, precisely tuning their frequency offset, detecting the beat signal with a photodetector, and analyzing it using an electrical spectrum analyzer.	1	High-resolution and high-sensitivity Strict requirements for reference light sources	0.6 Hz [228] 40 mHz [229] 100 Hz [230]
Brillouin stokes optical beating method	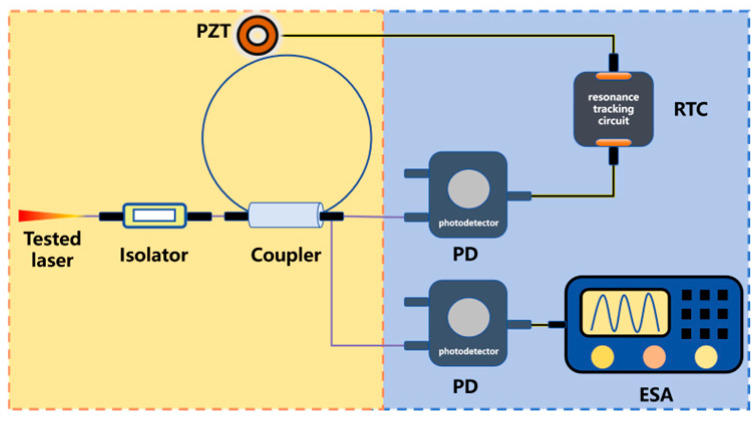	This technology measures the laser linewidth by using second-order Stokes waves in a fiber resonator as intrinsic references, generating same-direction second-order Stokes via pump excitation, coupling for beat frequency, analyzing FWHM of the spectrum, and controlling resonator length with resonant tracking and piezoelectric ceramics.	2	High-resolution Simple system (only one laser needed) Sensitive to environment Beat signal frequency range limitation due to large Brillouin shift	4.2 kHz [231] 100 kHz [232] 300 Hz [233] 5.5 kHz [234] 860 Hz [235] 3.84 kHz [236]
Delayed self-homodyne method	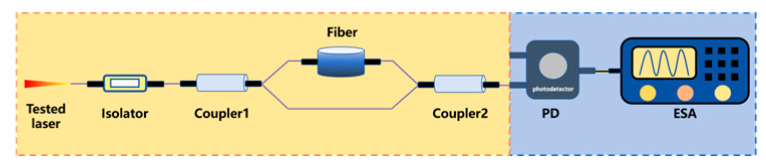	This technology uses an unbalanced Mach-Zehnder interferometer architecture to split the tested laser into a time-delayed signal via a fiber delay line and a reference signal, generating an optical beat signal through coherent interference, converting it into an electrical signal, and extracting the laser linewidth by analyzing the PSD of the electrical signal.	1	Avoiding dependence on the reference laser Zero frequency interference	18 MHz [237] 460 kHz [238]
Delayed self-heterodyne method	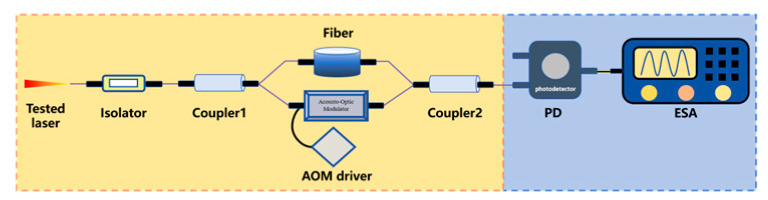 Mach-Zehnder interferometer-based delayed self-heterodyne method	The tested laser is split at Coupler1. One arm incorporates a delay fiber, to induce controlled decoherence, while the other arm routes light through an AOM generating a frequency shift. The obtained delayed beam and frequency-shifted beam are coupled at Coupler2 and detected by PD. Finally, ESA is used to collect data and display the detected signal.	1	Avoiding interference from zero frequency Long fiber length can introduce 1/f noise	120 kHz [239] 2.58 kHz [240]
	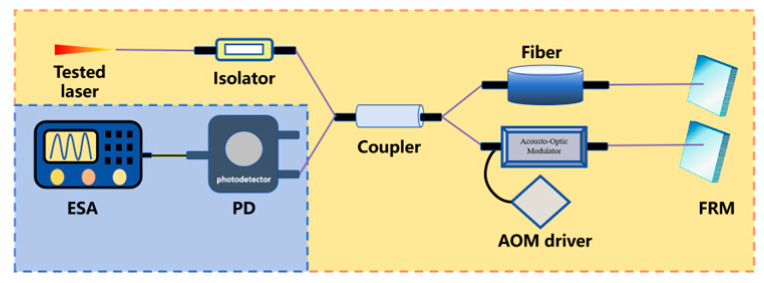 Michelson interferometer-based delayed self-heterodyne method	The system splits the tested laser via a coupler, introducing Faraday Rotator Mirror (FRM) to stabilize polarization and reduce noise. After passing through delay fiber, the laser is reflected by FRM, redoing the optical path, then interferes at the coupler for 2 times the fiber-length delay. Finally, PD detects and ESA acquires data.	1	FRM is introduced, maintaining the stability of polarization state and reducing the noise caused by random polarization state drift. Long fiber length can introduce 1/f noise.	[241]
	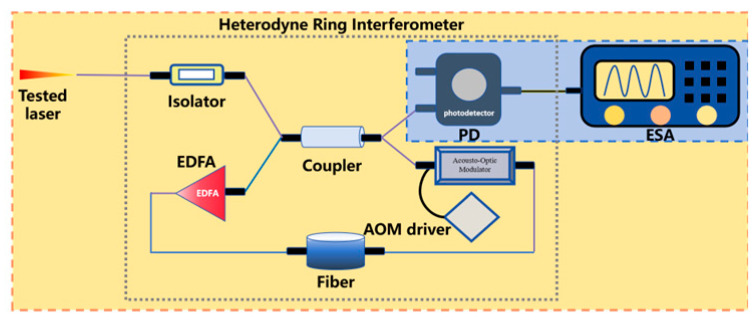 Gain compensation loop delay self-heterodyne method EDFA: erbium-doped optical fiber amplifier	This method uses a fiber ring’s multipass transmission to amplify delay time, reducing required delay fiber length. Resolving beams with different circulation cycles from the fiber ring enables acquiring theoretically infinite photocurrent spectral lines via ESA, with its architecture multiplying temporal delay between beams.	1	Can measure wide range of laser bands Sensitive to the environmental noise Unable to eliminate the impact of 1/f noise	680 Hz [242]
	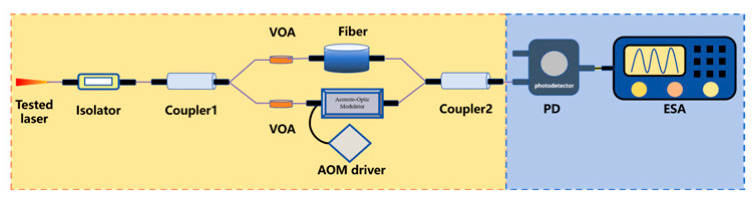 High-coherence envelope self-coherence detection VOA: Variable Optical Attenuator	This approach introduces two variable optical attenuators into the modified MZI-based delayed self-heterodyne method system. By establishing a quantitative mapping relationship between the contrast difference between the second spectral peak and the second trough (CDSPST) and the laser linewidth, the linewidth is calculated.	1	suppressing the spectral broadening induced by 1/f noise in the Gaussian linewidth of laser output	150 Hz [243,244] 98 Hz [245] 609 Hz [246]
	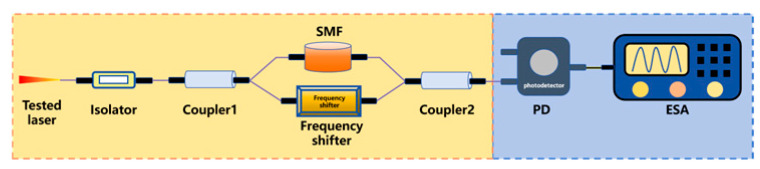 Dual-parameter acquisition (DPA) method SMF: Single-Mode Fiber	This method transforms conventional Lorent-zian fitting based on incoherent interference into dynamic modeling utilizing partially co-herent interference. By extracting the power difference between adjacent extrema in the first-order sidelobe and the frequency deviation between the central frequency and the zeroth-order minimum, the linewidth is measured.	1	No precise delay fiber length data needed	458 Hz [247]
	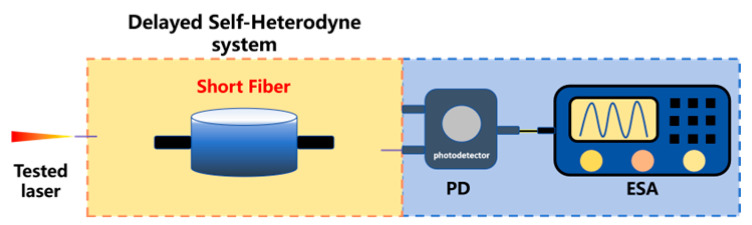 Linewidth measurement based on short-fiber delayed self-heterodyne	In order to completely eliminate the influence of 1/f noise from the root, a linewidth measurement method using short optical fibers for delayed autoheterodyne has emerged in recent years. When the delay fiber is short, the broadening of the measured power spectrum can be effectively suppressed.	1	eliminate the influence of 1/f noise	151 Hz [248] 944 Hz [249,250] 8 kHz [251] 2.53 kHz [252] 6.1 kHz [253,254] 1.753 kHz [255] 100 Hz [256]

The limitations of heterodyne detection methods primarily manifest in two aspects: beat signal fidelity and systemic noise contamination. First, during beat signal generation, spectral broadening models exhibit systematic deviations from actual linewidths due to compounded effects including fiber nonlinearities, harmonic distortion in photoelectric conversion, and quantization noise aliasing. Second, the frequency-domain characteristics of laser phase noise fundamentally arise from the superposition of spontaneous emission-induced 1/f noise and white noise, which dominantly governs lineshape broadening.

Two-beam interferometry, reliant on interference between independent laser sources, theoretically enable direct linewidth characterization through heterodyne signatures. However, frequency drift and relative intensity noise in reference lasers introduce uncontrollable errors. The Brillouin Stokes optical beating method exploits stimulated Brillouin scattering nonlinearities to map the frequency difference between pump and Stokes waves into measurable electrical signals. Nevertheless, pump power fluctuations and phonon relaxation time uncertainties exacerbate low-frequency noise. Delayed self-homodyne/heterodyne methods circumvent external reference interference through extended fiber delay lines in split-path configurations, yet respectively suffer from zero-frequency ambiguity and 1/f noise amplification.

**Figure 8:**
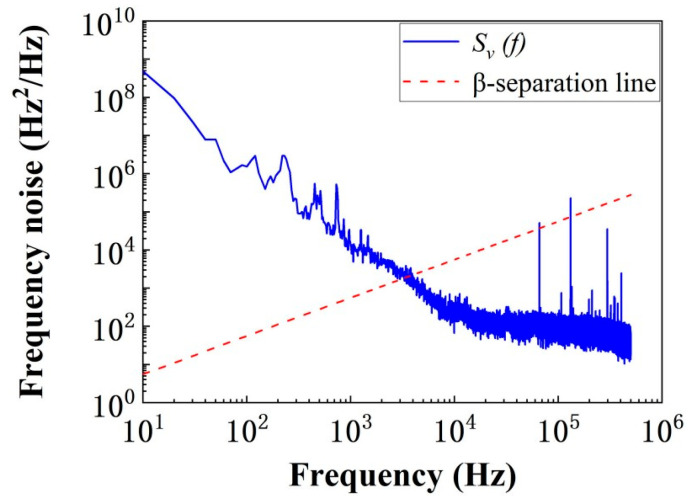
Frequency noise distribution and β-separation line [260].

To solve these predicaments, Elliott et al. were among the first to provide the theoretical relationship between laser line shape and frequency-noise PSD. Then the researchers put forward the β-separation theory which showed the β-separation line divides the frequency noise spectrum into two distinct regions [257], as illustrated in Figure 8. The first region above the β-separation line (*S_υ_(f)* > 8ln(2)*f/*π^2^) corresponds to the low-frequency modulation domain, which is dominated by 1/f noise, fundamentally determining the intrinsic linewidth of the measured laser. The other region below the β-separation line (*S_υ_(f)* < 8ln(2)*f/*π^2^) represents the high-frequency modulation domain characterized by white noise, resulting in a Lorentzian lineshape [258]. In this regime, the laser linewidth is exclusively governed by the spectral density of frequency noise [259].

In fact, the noise components in the first region with spectral density higher than its Fourier frequency (*S_υ_(f)* > *f*) generate Gaussian autocorrelation functions. The Fourier transform of the product of these autocorrelation functions yields the laser line shape. And this line shape is a Gaussian function, whose variance is the sum of the contributions of all high-modulation index noise components. Therefore, we can obtain a good approximation of the laser linewidth through the following simple expression [261]:

(13) $$FWHM=(8\ln(2)A{)}^{1/2}$$

(14) $$A=\int_{\frac{1}{T_{0}}}^{\infty }H(S_{\upsilon }(f)-8\ln(2)f/\pi^{2})S_{\upsilon }(f)df$$

where *H(f)* denotes the unit step function, A represents *S_υ_(f)* above the β-separation line in the frequency noise spectrum, and *T*_0_ signifies the measurement duration.

Overall, the β-separation theory provides a theoretical basis for linewidth measurement at complete Fourier frequencies. Based on the β-separation theory, researchers have proposed various measurement methods based on phase noise. This section systematically examines four indirect approaches corresponding to Route B in Figure 6: the linewidth measurement method based on cross-correlation and the β algorithm, the frequency discrimination method, the optical coherent reception method based on an interferometer, and the optical coherent reception method based on delayed self-homodyne/self-heterodyne techniques.

The linewidth measurement method based on cross-correlation and the β algorithm integrates mature radio-frequency cross-correlation frequency noise characterization [262] with β full-frequency demodulation; the former suppresses system noise via iterative operations, the latter enables full-band linewidth calculation through frequency noise integration [263,264,265]. Specifically, the laser under test first coherently beats with two reference sources; cross-correlation of the two beat signals eliminates noise to obtain the phase-noise autocorrelation function. Fourier transform yields the phase-noise power spectrum, and subsequent data conversion with β algorithm processing derives the laser linewidth.

The frequency discrimination method measures frequency noise by converting laser frequency fluctuations into intensity variations via a discriminator [266]. Its system includes a quadrature servo control to dynamically adjust laser frequency, maintaining the bias point in quadrature for maximum sensitivity and stabilizing frequency by compensating drift through feedback. This method enables comprehensive noise analysis [267,268] and avoids low-frequency noise by locking to the quadrature point [269] or shifting to the radio frequency domain via self-heterodyne detection [270]. However, PD conversion introduces extra noise; its accuracy depends on the discriminator, and the laser must operate in a narrow frequency range, limiting achievement of the ultra-low noise floor required for millihertz-linewidth laser characterization.

Optical coherent reception based on an interferometer determines the linewidth by analyzing the relationship between the phase noise of the interferometer and the frequency noise power spectrum of the laser [259]. Its distinctive feature lies in the adoption of a phase-difference interferometer system with a 3×3 coupler to measure differential phase information. The three output ports generate split beams with equal amplitude and 120° phase differences. After the signals are collected by an analog card, the phase noise PSD is obtained by analyzing the deviation of frequency-phase characteristics from the linear fitting curve and demodulating the phase information. The laser linewidth is then calculated using the β algorithm.

The optical coherent reception method based on delayed self-homodyne/self- heterodyne realizes the effective demodulation of signal light and local oscillator light by introducing an incoherent-to-coherent receiver (ICR) [69,271,272,273]. The laser under test and the delayed laser, after passing through a polarization controller, enter the ICR with a 90° mixer and are mixed with the local oscillator light, generating in-phase/quadrature (I/Q) signals. These I/Q signals are detected by a balanced photodetector (BPD), converted into electrical signals, and then amplified by a transimpedance amplifier (TIA). The amplified signals are sent to a digital signal processor (DSP) to extract phase noise information, and finally, the linewidth of the laser under testing is calculated. Table 2 presents the measurement devices, their descriptions and main features and some references.

**Table 2:** Four indirect measurement methods Based on Phase Noise [159,160,265,274,275,276,277].

	Measuring Equipment	Description	Main Hallmarks	Reference
Cross-correlation method and β algorithm	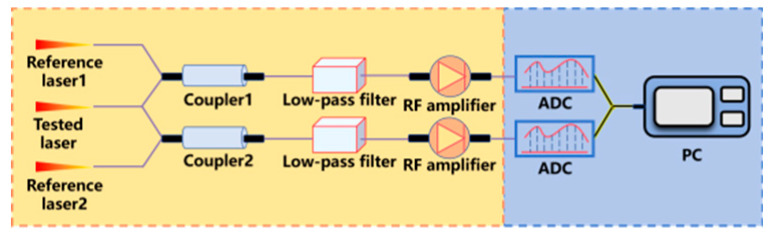 ADC:Analog-to-Digital Converter	The innovation of this methodology lies in the integration of the radio-frequency-domain cross-correlation frequency noise characterization technique with the β full-frequency-domain demodulation algorithm. The cross-correlation method iteratively suppresses system noise through multiple computational operations, while the β-algorithm achieves full-bandwidth linewidth resolution via frequency noise integration.	Avoiding linear fitting Suitable for any noise Complex calculation	26.9 kHz [159]
Frequency discrimination method	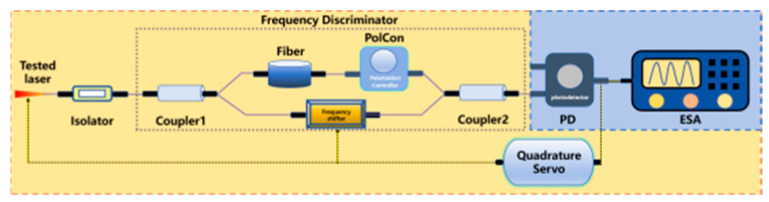 Polarization Controller: PolCon	Frequency discrimination is to characterize frequency noise by changing laser frequency fluctuations into intensity variations via a frequency discriminator, where the resulting intensity changes are monitored to derive frequency deviations. A quadrature servo control adjusts laser frequency dynamically via discriminator feedback, maintaining quadrature operation for optimal sensitivity and compensating drift for frequency stabilization.	Avoiding interference from zero frequency Frequency range is limited Precise control is necessary	0.7 Hz [160] 269 Hz [274] 45 Hz [275]
Optical coherent reception method	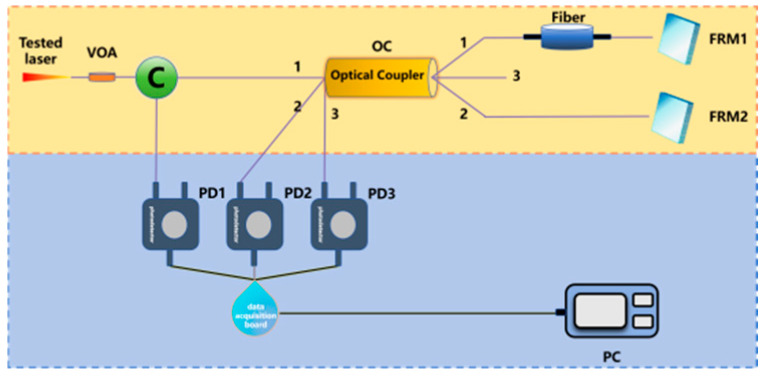 Based on an 120° interferometer	This method determines laser linewidth by establishing the quantitative relationship between interferometric phase noise and the laser’s frequency noise PSD. After signal collection via an analog card, the phase noise PSD is obtained by analyzing frequency-phase characteristics and linear fitting curve deviation from linear scanning, demodulating phase information, and calculating the laser linewidth using the β algorithm.	Measure instantaneous phase change directly Complex structure High cost	4.36 kHz [265] (minimum integrated linewidth) 3.58 kHz [265] (minimum Lorentzian linewidth)
Optical coherent reception method	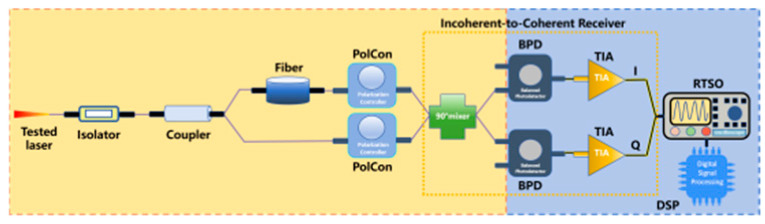 Based on delayed self-homodyne/self-heterodyne	The tested laser is split via a coupler into two beams. One propagates directly, while the other undergoes a time delay. Both beams are then routed through a PolCon before entering the incorporating an ICR for generating I/Q signals. The amplified signals are fed into a DSP system to extract phase noise characteristics, ultimately computing the linewidth.	Measure instantaneous phase change directly Complex structure High cost	50 kHz [276] 20 kHz ~ 2 MHz [277]

## 6. Linewidth Measurement Method Based on Electronic Information Processing

In the field of laser linewidth characterization, two conventional methodologies relying on beat-note power spectral analysis and phase noise evaluation have undergone continuous refinement and innovation, giving rise to diverse novel measurement approaches. These advancements not only enhance measurement precision and efficiency but also broaden applicability, enabling more accurate assessment of narrow-linewidth laser characteristics.

### 6.1. Frequency Comb-Based Method

This technique employs heterodyne beating between the emission frequency of the tested laser and the harmonic components of a frequency comb’s repetition rate. A voltage-controlled oscillator (VCO) tracking the beat frequency converts its frequency fluctuations into voltage signals, which are subsequently analyzed through feedback-controlled spectral measurements to derive laser linewidth.

M. Ravaro et al. [278] reported a method for measuring the intrinsic linewidth of terahertz quantum cascade lasers (QCLs) using near-infrared frequency combs. The QCL and frequency comb beams are collinearly focused onto a ZnTe crystal. A tandem configuration of waveplates and a polarizing beam splitter behind the crystal forms an ultrafast near-infrared electro-optic amplitude modulator driven by the terahertz electric field. The balanced detection output is frequency-shifted to match the operational frequency of the VCO via an RF synthesizer. For demodulation, a “tracking oscillator” technique is implemented, where the VCO converts beat frequency fluctuations into voltage signals. A fast Fourier transform analyzer measures the PSD of the output voltage, from which the frequency noise spectral density of the QCL is derived using the calibrated VCO sensitivity. At an output power of 2 mW, this system achieved an intrinsic linewidth measurement of 230 Hz.

This method is based on generating a difference frequency signal between the QCL frequency and the repetition frequency of the near-infrared optical frequency comb [279,280]. It enables the measurement of the frequency noise spectral density of terahertz QCLs at any frequency demonstrated to date. However, it is limited by the constraint that the emission frequency must fall within the comb spectrum bandwidth.

### 6.2. Optical Feedback Interferometry-Based Method

Compared to conventional interferometric methods, laser self-mixing techniques offer significant advantages by eliminating external detectors and simplifying optical alignment processes [281].

The tested laser is collimated via a lens and focused onto a planar mirror mounted on a piezoelectric actuator, typically driven by sinusoidal voltage modulation. A VOA ensures optimal feedback levels. Light reflected from the mirror interferes with the intracavity optical field, generating self-mixing signals, which are detected by a PD integrated at the laser rear facet, amplified, and digitized using a digital oscilloscope. Linewidth estimation is achieved through statistical analysis of interference fringe period histograms, combined with repeated root-mean-square phase noise measurements at varying target distances. M.C. Caldiroli et al. [282] implemented this methodology with an adjustable pinhole for dynamic feedback regulation, successfully characterizing a mid-infrared QCL with a measured linewidth of 280 kHz.

Compared with delayed self-homodyne and self-heterodyne methods, this method avoids extra external detectors and simplifies optical processing. Yet it has drawbacks: inability to distinguish individual noise sources (only fringe-period-averaged integrated linewidth); optical feedback-induced residual narrowing leading to narrower measured linewidths; thermal effects reducing the laser’s tuning coefficient (declining above 1 kHz [283]), with smaller coefficients reversing high-frequency fluctuations and causing underestimated linewidths.

### 6.3. Power Area Method (PAM)

The β-separation line method faces limitations in determining linewidth when confronted with complex frequency noise PSD profiles. PAM effectively resolves this challenge by providing estimation errors below 7% for both white noise and flicker frequency noise across nearly all measurement durations, while retaining the simplicity and intuitiveness of the β-separation line approach. Notably, PAM is applicable not only for determining laser linewidth but also for characterizing delayed self-heterodyne beat-note spectral linewidth. Zhou et al. [284] successfully employed this method to estimate linewidth in both delayed self-heterodyne and delayed self-heterodyne beat-note signals.

This method and theory may be applicable and useful in the applications of narrow-linewidth lasers, such as coherent optical communication, high-resolution spectroscopy, and optical frequency combs.

These three types of methods differ from traditional laser linewidth measurement approaches that rely on optical processing. Instead, they optimize laser linewidth measurement methods by leveraging modern electronic information processing techniques (e.g., detection via voltage signals) and algorithm optimization. Furthermore, these methods are complementary in their technical routes; the frequency comb method focuses on improving precision, optical feedback interferometry emphasizes system simplification, and the power area method is dedicated to algorithm optimization.

## 7. Comparative Analysis of Laser Linewidth Measurement Techniques

**Figure 9:**
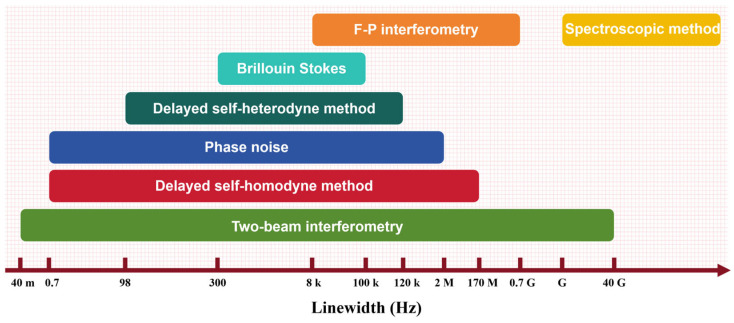
Measurement Ranges of Laser Linewidth Characterization Methods.

Figure 9 illustrates the measurement ranges of reported laser linewidth characterization methods. Selection of an appropriate methodology requires comprehensive consideration of laser performance parameters and available experimental infrastructure to ensure optimal measurement outcomes.

## 8. Conclusions and Perspectives

In summary, laser linewidth, an essential parameter for evaluating spectral purity and coherence, plays a pivotal role in advancing photonic technologies across diverse domains, including optical communications, quantum computing, and precision sensing. Existing linewidth measurement techniques can be broadly categorized into two regimes: wide-linewidth methods and narrow-linewidth methods. For wide-linewidth lasers, GHz-range spectroscopic methods and MHz-range F-P interferometry offer relatively mature solutions, with numerous commercial instruments readily available. For narrow-linewidth lasers, direct and indirect methods have been developed. Direct approaches rely on signal power spectral analysis and include techniques such as two-beam interferometry, Brillouin Stokes optical beating, delayed self-homodyne, and delayed self-heterodyne methods. Indirect approaches, which rely on phase noise analysis, include the linewidth measurement technique based on cross-correlation and the β-separation line algorithm, the frequency discrimination method, interferometer-based optical coherent reception, and optical coherent reception schemes employing delayed self-homodyne or self-heterodyne techniques.

At present, the convergence of photonic integration, quantum technologies, and artificial intelligence is accelerating the advancement of laser linewidth metrology toward sub-Hz precision, superior noise immunity, and intelligent real-time operation. While substantial progress has been achieved, the continued development of accurate, robust, and low-noise measurement techniques remains critical to support the evolving demands of next-generation ultra-narrow-linewidth laser systems. In particular, the rapid progress of artificial intelligence is propelling linewidth measurement systems toward greater autonomy, adaptability, and interpretability, enabling advanced data analytics, real-time system calibration, and predictive diagnostics. These innovations are expected not only to support the advancement of fundamental scientific instruments such as atomic clocks and gravitational wave detectors, but also to transform laser applications ranging from ultra-fine precision to ultra-wide fields of view, and from short-range to ultra-long-distance scenarios.

## Data Availability

Data underlying the results presented in this paper are not publicly available at this time but may be obtained from the authors upon reasonable request.
